# Chilean Darwin Wasps (Ichneumonidae): Biogeographic Relationships and Distribution Patterns

**DOI:** 10.3390/insects15060415

**Published:** 2024-06-04

**Authors:** Diego G. Pádua, Andrés Moreira-Muñoz, Vanezza Morales-Fierro, Rodrigo O. Araujo

**Affiliations:** 1 Laboratorio de Entomología General y Aplicada, Centro de Investigación de Estudios Avanzados del Maule, Universidad Católica del Maule, Avenida San Miguel, 3605, Talca 3460000, Chile; paduadg@gmail.com; 2 Instituto de Geografía, Pontificia Universidad Católica de Valparaíso, Avenida Brasil, 2241, Valparaíso 2340025, Chile; andres.moreira@pucv.cl; 3 Herbario EIF & Laboratorio de Evolución y Sistemática, Facultad de Ciencias Forestales y de la Conservación de la Naturaleza, Universidad de Chile, Av. Santa Rosa, 11315, La Pintana, Santiago 8820808, Chile; vdmorales@gmail.com; 4 Museo Nacional de Historia Natural, Interior Quinta Normal, s/n, Santiago 8350410, Chile

**Keywords:** biodiversity, Chile, cosmopolitan, endemism, faunistic elements, Holarctic, Neotropics, parasitoid wasps, richness

## Abstract

**Simple Summary:**

The global biodiversity crisis poses many challenges for humanity, and continuing to classify and gain knowledge of the “hidden biodiversity” of less studied groups considered hyper-diverse insect groups, such as the parasitoid wasp (Ichneumonidae), is one of them. There is a particular need for more taxonomic and distributional knowledge of the Neotropics and its neighboring regions. We assessed the current knowledge of the Ichneumonidae, otherwise known as Darwin wasps, in Chile, a country with a diverse ecogeographic gradient, from the southern cold and humid tip of South America approaching the Antarctic Circle to the arid Atacama beyond the Tropic of Capricorn. Following the most up-to-date taxonomic knowledge, we assessed biogeographic relationships at the genus level and the spatial patterns of biodiversity at the species and genera levels along the latitudinal gradient. The results show that biogeographic relationships are based on six faunistic elements (cosmopolitan; endemic; Neotropical; Holarctic–Oriental; south-temperate; and Australasian), reinforcing the early view of two different areas for Chilean ichneumonids: a northern province and a Neantarctic realm, with a high percentage of endemic genera and species. Spatial biodiversity patterns assessed at different scales show a bimodal distribution of richness: around 34° and 38° S in the Metropolitan and Araucanía Regions. From an ecoregional perspective, richness is concentrated in the Valdivian temperate forests, but when assessed at a 0.5 × 0.5 cell scale, several outstanding cells are in the contact zone between the temperate forests and the Chilean Matorral in the Central Chilean biodiversity hotspot. Interpreting richness involves two phenomena difficult to disentangle: genuine species diversity and collection bias closer to areas with larger human populations. In contrast, the Atacama Desert shows little or no presence of Darwin wasps, which is to be expected due to the lack of potential hosts. These results reinforce the need to continue sampling and studying available collections to help close the knowledge gaps already recognized as Wallacean and Linnean shortfalls in order to gain additional information on potential threats to endemic genera and species.

**Abstract:**

Ichneumonidae, or Chilean Darwin wasps, are an important component of South American hymenopteran diversity, but the taxonomic and distributional knowledge on this insect is still deficient. Taking advantage of recently updated taxonomic knowledge, we assessed biogeographic relationships at the genus level and biodiversity spatial patterns along the latitudinal gradient. The results show the presence of 264 species in Chile, arranged in 102 genera and 22 subfamilies. Biogeographic relationships are based on six elements (cosmopolitan (n = 50; 36%), endemic (n = 29; 21%), Neotropical (n = 22; 16%), Holarctic–Oriental (n = 19; 14%), south-temperate (n = 16; 11%) and Australasian) and composed of just three genera: *Anacis*, *Labena*, and *Meringops*. Species and genera show a bimodal distribution along the latitudinal gradient: around 34° and 38° S. From an ecoregional perspective, richness is concentrated in the Valdivian temperate forests, but when assessed at a 0.5 × 0.5 cell scale, several outstanding cells are in the contact zone between the temperate forests and the Chilean Matorral. On the other hand, the Atacama Desert shows little or no presence of Darwin wasps. The results agree with Charles Porter, who identified a northern province composed of Neotropical and cosmopolitan genera with their own representatives in the far north (11 genera), a distributional gap in the core of the Atacama Desert, and around 128 genera in Porter’s Neantarctic realm, covering all of Chile from 25° S to Cape Horn, including the Juan Fernandez islands. These results reinforce knowledge gaps and the need for more sampling and studies of available collections. Due to sampling gaps at this stage, identifying a continued increase or decrease in richness towards higher latitudes is not possible. More taxonomic and distributional information is also needed to assess potential threats to endemic genera and species.

## 1. Introduction

**Figure 1:**
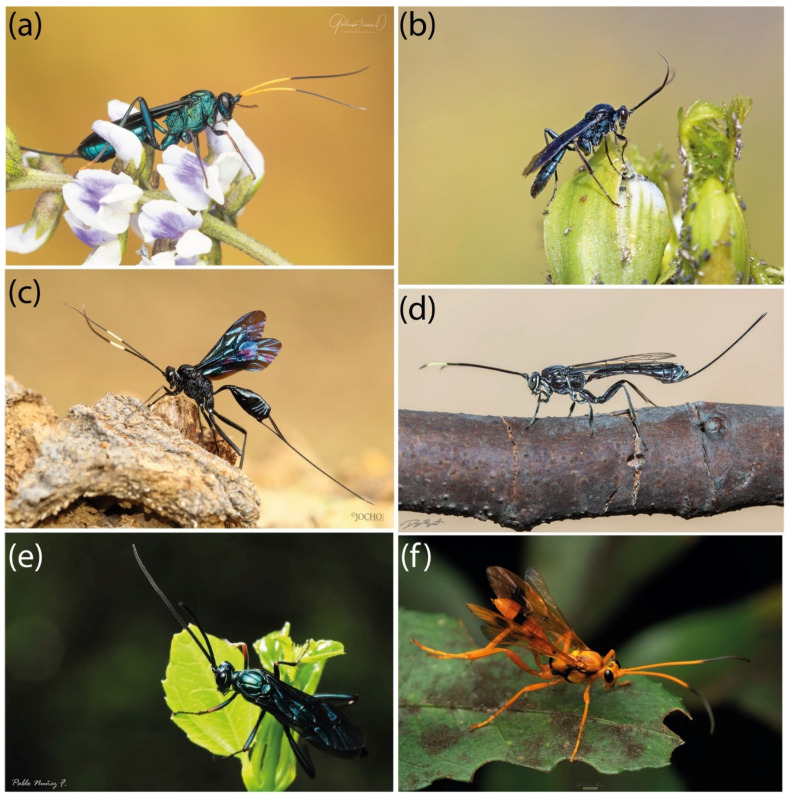
Darwin wasp representatives from Chile (in vivo): (**a**) *Trachysphyrus* sp. (Cryptinae); (**b**) Ichneumoninae sp.; (**c**) *Dotocryptus* sp. (Cryptinae); (**d**) *Macrogrotea* sp. (Labeninae); (**e**) *Trachysphyrus* sp. (Cryptinae); (**f**) *Hoplismenus* sp. (Ichneumoninae).

If we take a close look at the bark of a tree in a subtropical forest, we will have a high likelihood of finding a Darwin wasp (Ichneumonidae) (Figure 1) in the struggle to secure a host for its eggs. Ichneumonidae are parasitoid wasps, a major component of global biodiversity, and at the same time a poorly studied group [1]. They comprise the most speciose group of Hymenoptera (one of the most species-rich orders on Earth), participate in a wide range of ecological processes, and provide humanity with essential ecosystem services [2]. Nevertheless, there are various impediments to improving our knowledge of the diversity and functional roles of parasitoid wasps, such as the Linnean shortfall (most species have not been described) and Wallacean shortfall (the distribution of many described species is unknown) [3].

These various impediments have dramatic consequences for insect conservation [4,5], especially actions to protect and manage so-called “hidden biodiversity” [6], and apply especially to (a) laboriously identifiable species; (b) those with unknown socio-economic potential; and (c) those which can only be collected in areas difficult to access [7]. The knowledge gaps related to parasitoid wasps and their systematic underestimation “biases our understanding of multi-trophic tropical interactions and determination of large-scale biodiversity patterns” [7] (p. 4697).

The lack of specialized entomologists and accurate, standardized, and cost-effective sampling protocols are added impediments to the plight to advance our knowledge of parasitoid wasps [2].

Though accurate updated numbers are lacking, estimates suggest that Hymenoptera diversity in the Neotropics is greater than in the Nearctic, Palearctic, and Australian regions [8].

Ichneumonidae are the largest Hymenoptera family, and are currently divided into 42 subfamilies [9] with over 25,000 valid species [10]. Besides having the highest endemism rates, this family is also highly relevant for the practice of biological control since the species that comprise it obligatorily deposit their eggs in arthropods [8].

The Chilean biota has attracted much attention due to its connection with the Neotropics on the one hand, and its connection with Australasia as a remnant of an ancient Gondwanan biota on the other [11]. Indeed, the long latitudinal gradient and the presence of different environments along this gradient make Chile a biogeographic laboratory. The rapid uplift of the Andes since the Late Miocene prompted the isolation of biota, leading to remarkable levels of endemism [11].

Regarding species richness, in the northern hemisphere, there are indications of an inverse richness pattern; that is, an increase in richness towards higher latitudes. In the southern hemisphere, the lack of complete inventories creates difficulty in testing general-to-regional distribution patterns, and the richness of several groups tends to be concentrated in mid-latitudes. This pattern still needs to be tested regarding several explanations related to life history traits and attack strategies, mainly divided into different ovipositor lifestyles: idiobionts and koinobionts [12,13].

Chile has a very unique Ichneumonidae fauna, with differences in comparison to the Neotropics (see [14,15,16]). Porter [15] mentions that Chilean Darwin wasps differ significantly from the rest of South America and cannot be included as a sub-element of the Neotropical, due to “its exceptionally high number of endemic genera and its surprisingly few Neotropic genera for an area in geographic proximity to the American tropics” [15] (p. 38).

Indeed, Ichneumonidae are the family with the most endemic genera and species in Chile (including one endemic subfamily: Claseinae). The last published catalog reports a total of 36 endemic genera and 170 endemic species, out of a total of 88 genera and 193 recorded species [17]. The Chilean fauna includes representatives of cosmopolitan, Holarctic or Holarctic–Oriental, Neotropical, Andino-Patagonian, and Transantarctic (sharing species with Australia and New Zealand) genera, in addition to some genera that are widely but disjunctively distributed in both the Northern and Southern Hemispheres [15].

Porter [15] hypothesizes that the aberrant and endemic Chilean Darwin wasp fauna probably represents survivors that moved north from Antarctica before the glaciation and that evolved in isolation for the last 40 million years due to mountains, desertification, and a cold climate affecting the region’s eastern and northern boundaries by the mid-Cenozoic.

Thorough research into Chilean ichneumonid fauna ecology is crucial to understanding interspecies interactions and distribution patterns. The main goal is to pinpoint endemic areas for the conservation and investigation of native species for the effective biological control of agricultural pests.

We took advantage of the most up-to-date taxonomic revision of the Chilean Ichneumonidae, allowing us to make progress towards two specific goals: (a) disentangling the biogeographic relations of Chilean Darwin wasps at the genus level; (b) discovering the spatial patterns of biodiversity along the latitude and altitude gradients.

## 2. Materials and Methods

### 2.1. Study Area

**Figure 2:**
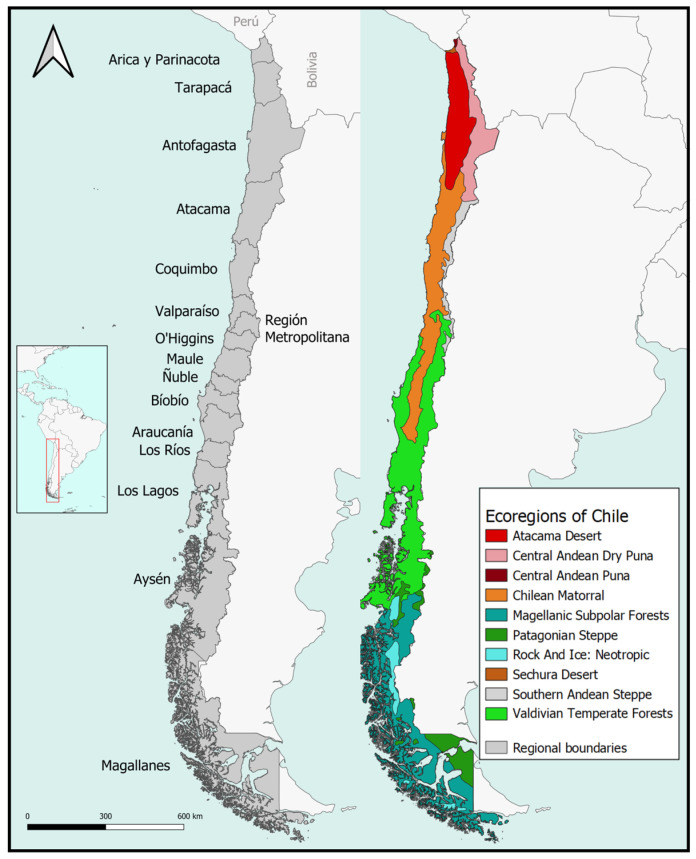
Study area: (**left**) administrative regions; (**right**) ecoregions according to Dinerstein et al. [18].

Due to its current geographical conditions, Chile is considered a biogeographic island, bordering the Sechura desert in Peru beyond the Capricorn Tropic to the north, the Andean highlands to the east, the Pacific Ocean to the west, and Cape Horn approaching the Antarctic Circle to the south. The latitudinal gradient spans from the northern dry areas of the most arid desert in the world, Atacama, toward subtropical scrubs, Mediterranean sclerophyllous forests, deciduous forests, and the temperate evergreen Valdivian Forest in the south. Further south are the subantarctic moors and dwarf forests of Magallanes approaching Cape Horn. This high diversity in environments and their temporal evolution give insects like hymenopteran, coleopteran, and other diverse groups opportunities to diversify, resulting in a high proportion of endemic species and genera. A useful map of Chilean environments, suitable for continental comparisons, is the map of ecoregions by Dinerstein et al. [18]. The main ecoregions in the country from north to south are the Atacama Desert, Chilean Matorral, Valdivian temperate forests, and the Magellanic subpolar forests. On the borders with Argentina and Peru, we also have representation of the Sechura Desert, the Central Andean dry Puna, the Southern Andes Steppe, and the Patagonian Steppe. Taking these ecoregions as a base, we plotted the number of species and genera (Figure 2).

### 2.2. Biogeographic Relationships

For this analysis, 139 genera registered in Chile were considered according to Araujo et al. [19], except the introduced genera *Megarhyssa* and *Rhyssa*. We also included 35 genera with undetermined species (a total of 139 genera) (see Table S1). The genus *Stiboscopus* was not considered in the biogeographic classification because the genus is recorded by Porter [15] without species identification, and currently the genus has been divided into several genera, with *Stiboscopus* being synonymous with the genus *Lysibia*. It is also because we do not know which genus/genera the specimens belong to. The assessment of biogeographic relationships, including the classification of biotic elements, is a traditional task in biogeography [20,21,22,23]. We based our analysis on previous classifications of Chilean biota [11,15,24,25].

Porter [15], based on the knowledge available at the time, presented a fine description of biogeographic relationships among Chilean Ichneumonidae. He recognized 131 genera (including a couple undescribed) and arranged them in two main groups: (a) the genera restricted to the northern province, north of 25° S, including valleys in a desertic matrix and the high Andes adjacent to Peru and Bolivia; and (b) the Neantarctic realm south of 25° S encompassing all of central and southern Chile. Porter arranged 121 genera into five biogeographic elements in this Neantarctic realm: (a) endemic, (b) cosmopolitan, (c) Holarctic–Oriental, (d) Neotropical, (e) Australasian or Transantarctic, and (f) Holarctic–Neotropical–Australasian disjunct.

Following current taxonomic and distributional knowledge, we retrieved the following faunistic elements: (a) endemic, (b) cosmopolitan, (c) Holarctic–Oriental, and (d) Neotropical and Australasian. We added a specific element retrieved by Moreira-Muñoz [11] for Chilean flora: the south-temperate element, which encompasses Neotropical genera but occurs only south of 33° S in Chile and adjacent Argentina, mainly in temperate forests.

According to current knowledge, Porter’s Holarctic–Neotropical–Australasian disjunct element is composed only of the genus *Isdromas*, but this genus can easily be considered subcosmopolitan.

### 2.3. Biodiversity Spatial Patterns

For this analysis, we only considered genera with determined species (a total of 102 genera, according to Araujo et al. [19]).

Data cleaning included reviewing the geo-referencing of 939 individuals. We used geographic name repositories (Geonames and Mapcarta) and our own localities database. We had to disregard 2.7% of the data due to misspellings or incomplete distributional information (e.g., a whole region, confused names, etc.). After database cleaning and the erasure of duplicates coordinated in individual collections, our database consisted of 914 records, encompassing 264 species in 101 genera (Table S2).

Maps were generated on two scales using ArcGis 10.3, considering ecoregions and cells of 0.5 × 0.5 degrees. This cell size has been shown to perform well at a national scale compared to 1 × 1 degree, which is better for visualization at the level of administrative regions (Figure 2). This has no biological meaning but is informative for richness and collection efforts.

## 3. Results

### 3.1. Biogeographic Relationships

For this analysis, all genera documented in Chile (totaling 139 genera) were taken into account following Araujo et al. [19].

The biogeographic relationships at the genus level showed a remarkable presence of 50 cosmopolitan (36%) and 29 endemic genera (21%) (Table 1). Porter [15] has already noted that the proportion of endemic genera is similar to or greater than Madagascar, New Zealand, and Australia [22]. Endemic genera (Neantarctic genera—sensu Porter) [14] are mostly represented by one unique species collected in a few localities.

The other strong elements in the Chilean Darwin wasp genera are the Neotropical (n = 22; 16%), Holarctic–Oriental (n = 19), and south-temperate (n = 16) elements. The least represented element is the Australasian (Transantarctic in the sense of Porter [15]), with just three genera: *Anacis*, *Labena,* and *Meringops* (see Section 4).

#### 3.1.1. Cosmopolitan Element

The cosmopolitan element of Chilean Darwin wasp genera is composed of 50 genera: *Habronyx*, *Parania*, and *Therion* (Anomaloninae); *Exetastes* and *Lissonota* (Banchinae); *Brachycyrtus* (Brachycyrtinae); *Campoletis*, *Campoplex*, *Casinaria*, *Diadegma*, *Dusona*, *Hyposoter*, *Meloboris*, and *Venturia* (Campopleginae); *Pristomerus* and *Trathala* (Cremastinae); *Cryptus* and *Mesostenus* (Cryptinae); *Diplazon*, *Syrphoctonus*, and *Woldstedtius* (Diplazontinae); *Diphyus*, *Eutanyacra*, *Hoplismenus*, *Ichneumon*, *Melanichneumon*, *Setanta*, *Dicaelotus*, *Tycherus*, and *Platylabus* (Ichneumoninae); *Cidaphus* and *Mesochorus* (Mesochorinae); *Colpotrochia* and *Hypsicera* (Metopiinae); *Enicospilus* and *Ophion* (Ophioninae); *Megastylus*, *Symplecis*, and *Stenomacrus* (Orthocentrinae); *Dichrogaster*, *Gelis*, *Xenolytus*, *Charitopes*, and *Atractodes* (Phygadeuontinae); *Clistopyga*, *Tromatobia*, *Itoplectis*, and *Pimpla* (Pimplinae); and *Netelia* (Tryphoninae). *Isdromas* (Phygadeuontinae) is distributed in Chile (Tarapacá region), Argentina, Brazil, Peru, and Ecuador in the Neotropics, Honduras in Central America, the United States in North America, and Australia. Hence, it can be considered subcosmopolitan (see Table S1). Indeed, most genera included in the element have a wide distribution in more than two continents or more than two main climatic zones (e.g., tropical and temperate). The element should be called *subcosmopolitan* (or semicosmopolitan, according to Porter [15]) because only half the genera occur in all continents, while the other half occur in the Nearctic, Oriental, Eastern and Western Palearctic, Neotropical, and European regions (Table S1).

#### 3.1.2. Endemic Element

The endemic element is composed of genera distributed in continental Chile and the Juan Fernandez Islands. There is one endemic subfamily (Claseinae) and 29 endemic genera: *Archoprotus* and *Valdiviglypta* (Banchinae); *Clasis* and *Ecphysis* (Claseinae); *Caenopelte*, *Araucacis*, *Nothischnus*, and *Periplasma* (Cryptinae); *Pedinopa*, *Cacomisthus*, *Petilium*, and *Stipomoles* (Ctenopelmetinae); *Barronia* (Eucerotinae), *Chilelabus*, *Ithaechma*, and *Zophoplites* (Ichneumoninae); *Gauldianus* (Labeninae), *Chineater*, and *Latilumbus* (Mesochorinae); *Pedunculus* (Pedunculinae), *Acidnus*, *Rhabdosis*, *Surculus*, *Peumocryptus*, *Bilira*, and *Teluncus*, (Phygadeuontinae); and *Notophrudus* (Tersilochinae).

These genera are distributed in Central Chile (*Archoprotus*, *Periplasma*, and *Chilelabus*), Central–Southern Chile (*Araucacis* and *Ithaechma*), and most predominantly in Southern Chile (*Valdiviglypta*, *Ecphysis*, *Caenopelte*, *Nothischnus*, *Clasis*, *Pedinopa*, *Cacomisthus*, *Petilium*, *Stipomoles*, *Barronia*, *Zophoplites*, *Gauldianus*, *Chineater*, *Latilumbus*, *Pedunculus*, *Acidnus*, *Rhabdosis*, *Surculus*, *Peumocryptus*, *Bilira*, *Teluncus*, and *Notophrudus*).

#### 3.1.3. Neotropical Element

The Neotropical element is composed of 22 genera, with Cryptinae the predominant one (*Dotocryptus Trachysphyrus Cyclaulus*, *Aeglocryptus*, *Cosmiocryptus*, *Hypsanacis*, *Itamuton*, *Neocryptopteryx*, *Phycitiplex*, and *Xylacis*), in addition to a few other genera of subfamilies such as *Cecidopimpla*, and *Diradops* (Banchinae); *Prochas* (Campoplegionae); *Coelorhachis* (Ctenopelmatinae); *Carinodes*, *Diacanthatius*, and *Thymebatis* (Ichneumoninae); *Alophophion* (Ophioninae), *Grotea* (Labeninae), *Calliephialtes*, and *Odontopimpla* (Pimplinae); and *Stethantyx* (Tersilochinae) (see Table S1).

#### 3.1.4. Holarctic–Oriental Element

The Holarctic–Oriental element is composed of *Glypta* (Banchinae), *Cymodusa*, *Campoctonus*, *Microcharops*, *Nemeritis*, and *Phobocampe* (Campopleginae); *Sussaba* (Diplazongtinae), *Scolomus*, and *Seticornuta* (Metopiinae); *Apoclima*, *Helictes*, and *Gnathochorisis* (Orthocentrinae); *Aclastus*, *Ethelurgus*, *Stilpnus*, and *Distathma* (Phygadeuontinae); *Stenobarichneumon* (Ichneumoninae); and *Liotryphon* and *Polysphincta* (Pimplinae).

#### 3.1.5. South-Temperate Element

The south-temperate element is composed of genera distributed in the Chilean Andes Mountains and adjacent Argentina. In Chile, 16 genera of Darwin wasps were found within this element: *Tatogaster* (Tatogastrinae); *Geraldus* (Banchinae); *Aglaodina*, *Chilecryptus*, *Myrmecacis*, *Oecetiplex*, *Picrocryptoides*, *Sciocryptus*, and *Xiphonychidion* (Cryptinae); *Cataptygma* and *Tetrambon* (Ctenopelmatinae); *Barythixis*, *Chilhoplites*, *Notophasma* (Ichneumoninae); *Torquinsha* (Labeninae); and *Lepidura* (Mesochorinae). All species of the genera classified under the south-temperate element have a Neantarctic distribution mainly in the Chilean part, and most south-temperate Darwin wasps with distribution in adjacent Argentina are present in Neuquén Province. The only exception could be *Aglaodina* (Cryptinae), reaching Antofagasta at 23°17′ south latitude.

*Petilium* and *Notostilbops,* classified as endemic, may in the future be classified as south-temperate, mainly due to the species distributed in Natales and Punta Arenas that are probably also in Argentina.

#### 3.1.6. Australasian Element

The Australasian element (Transantarctic in the sense of Porter [15]) is composed of genera distributed in Australia, South America, and the Pacific islands. In Chile, only three genera comprise this element: *Anacis* (Cryptinae), *Labena* (Labeninae), and *Meringops* (Phygadeuontinae).

### 3.2. Spatial Pattern of Biodiversity along the Latitudinal Gradient

**Figure 3:**
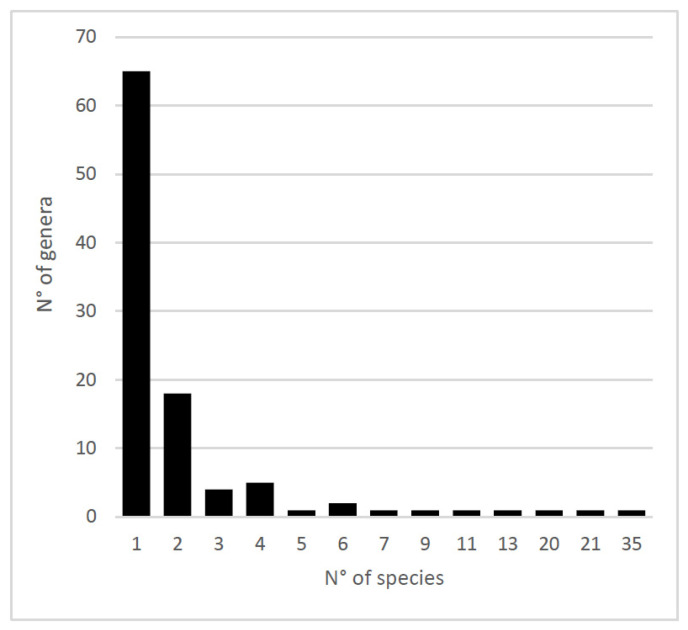
Number of species per genus among Chilean Ichneumonidae (only genera with determined species—n = 102).

For this section, we focused on genera with identified species as revised by Araujo et al. [19]. This database is composed of 922 records encompassing 264 species arranged in 102 genera. More than half of the genera (n = 65) are composed of only one species (Figure 3).

Regarding the assessment of ecoregions, the largest number of species and genera (including 65 monospecific genera) are in the Valdivian temperate forests (167 species and 66 genera). The second ecoregion is the Chilean Matorral (with 89 species and 48 genera). Both ecoregions together make up the Chilean Winter Rainfall–Valdivian Forests biodiversity hotspot [26,27].

**Figure 4:**
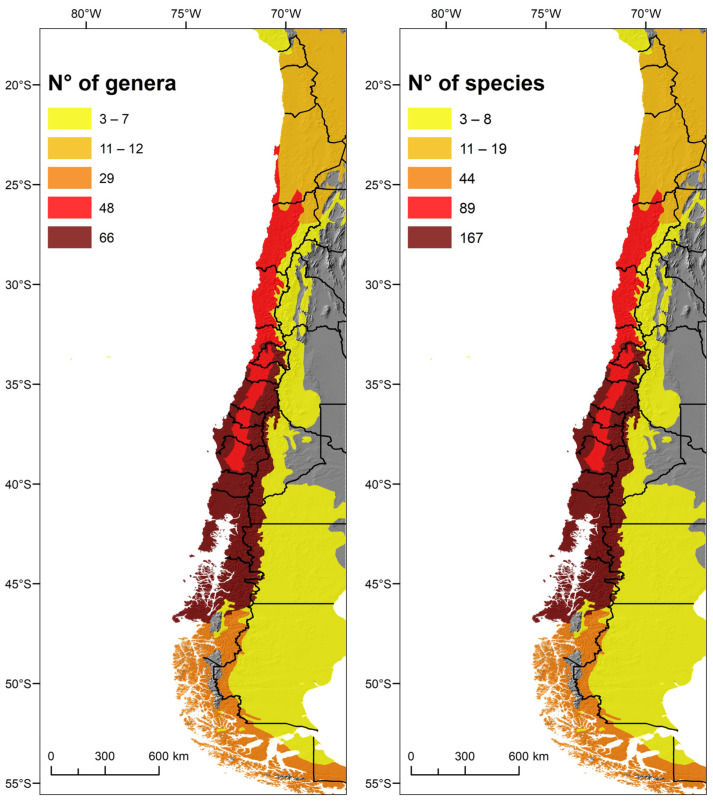
Ecoregions and species/genera richness. Ecoregions on the border with Argentina, Bolivia, and Peru only include data for Chile (only genera with determined species—n = 102).

**Figure 5:**
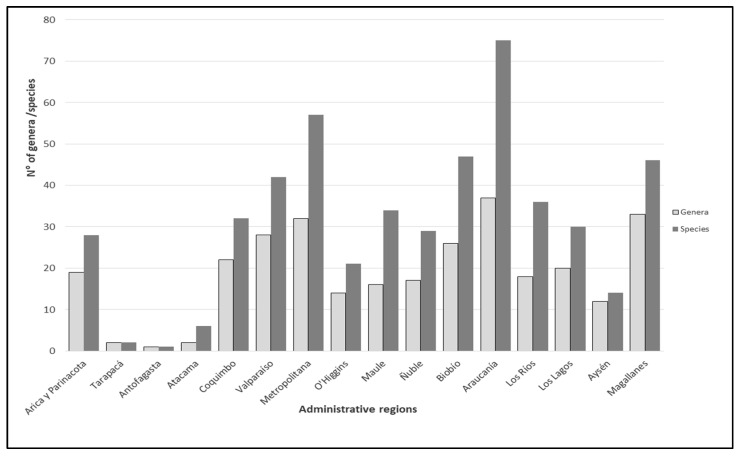
Species and genera richness for each administrative region (only genera with determined species—n = 102).

Regarding administrative regions following the latitudinal gradient, there is a bimodal distribution of species and genera richness: around 34° S in the Metropolitan Region and 38° S in the Araucanía Region (Figure 4 and Figure 5). The regions least suited for ichneumonid wasps are the arid northern regions, especially the Antofagasta Region at the center of the Atacama Desert (Figure 3, Figure 4 and Figure 5).

**Figure 6:**
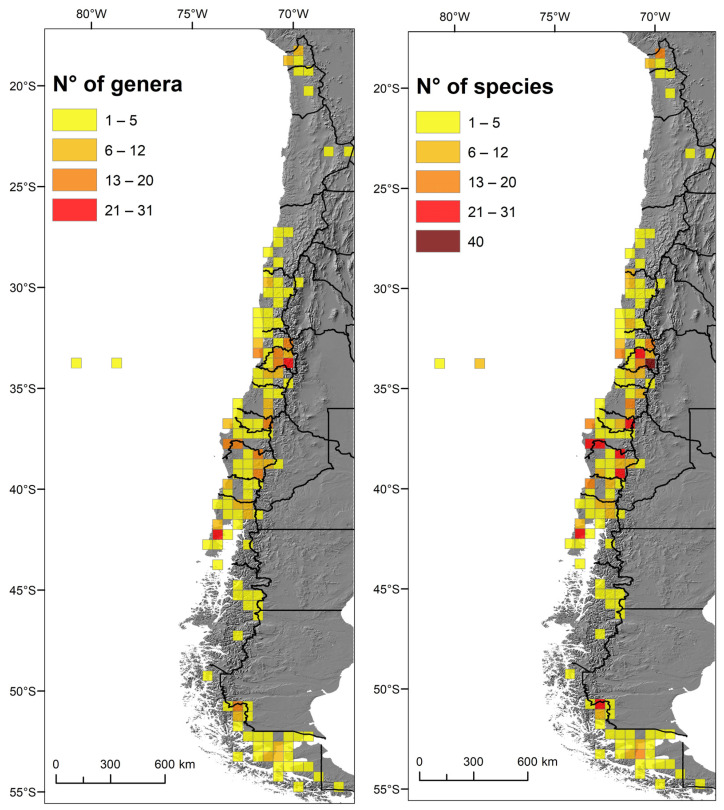
The richness of Chilean ichneumonid species plotted in 0.5 × 0.5 cells: (**left**) number of genera; (**right**) number of species.

Plotted in cells of 0.5 × 0.5 degrees, the concentration of ichneumonid biodiversity is highest in the Santiago Andes (Figure 6). Several cells in the Andes of the Maule, Ñuble, and Araucanía Regions, as well as the coast of Biobío and Chiloé, also stand out (Figure 6).

## 4. Discussion

Current estimates suggest that the Neotropics host the highest Hymenoptera diversity globally [8]. By modeling host–parasitoid systems, Forbes et al. [28] proposed that there may be 2.5 to 3.2 times more Hymenoptera species than Coleoptera. This is not just a clue for entomologists, but also useful from ethological and ecological perspectives. As potential biological pest control agents, and in their still little-known ecological roles, the importance of these estimates to insect conservation is enormous.

Regarding Chilean ichneumonids, our understanding is still limited in terms of taxonomic and distributional aspects (Linnean and Wallacean shortfalls, respectively), even with the recent availability of an updated catalog [19]. There are currently a total of 139 genera classified in 23 subfamilies [19]. This represents 37% more than the last authoritative catalog [17] and is closer to the numbers produced by Porter [15] over 3 decades ago. Porter considered the Chilean ichneumonid fauna to be composed of 131 genera and over 170 species (see also [29]). At the time, Porter increased taxonomic knowledge by 100% over previous studies. He considered that increasing taxonomic knowledge would result in the discovery of 1000 to 1500 species. We are still far from these numbers, but they are continuously increasing. Still, 35 genera recognized by Porter and considered valid for Chile lack any specimens classified at the species level. The stability of numbers at the genus level and the gaps at the species level respond to two possible phenomena: the lack of specialists to increase these numbers (i.e., identification, collection with specific traps, etc.), and the presence of a depauperate fauna in Chile, rich in endemism but not as rich in species numbers as in the rest of the Neotropics. The country’s prolonged biogeographic isolation accounts for the high proportion of endemism at the genus and species levels, but continuous environmental disturbances at a geological scale could also lead one to hypothesize innumerable extinctions [15].

The biogeographic relationships of Chilean ichneumonids have an intrinsic relationship to the evolution of Chilean biota as a whole, but one that is not well studied. Porter [15] arranged the Chilean genera in two main groups: (a) the genera restricted to the northern province, north of 25° S, including valleys in the Atacama Desert and the high Andes adjacent to Peru and Bolivia; and (b) the Neantarctic realm south of 25° S encompassing all of central and southern Chile.

According to this approach, the northern province is mainly composed of Neotropical and cosmopolitan genera, with their representatives in the far north shared with Peru and Bolivia, such as species from the genera *Brachycyrtus*, *Carinodes*, *Cosmiocryptus*, *Cyclaulus*, *Cymodusa*, *Hypsanasis*, *Isdromas*, *Itoplectis,* and *Mesostenus*. We can now add representatives of *Lissonota* and *Microcharops* to this list (one species each from the Azapa Valley).

All the other 120 genera belong to Porter’s Neantarctic realm, encompassing all of Chile south of 25° S toward Cape Horn, including the Juan Fernandez islands. The debate between the existence of a Neotropical realm and a Neantarctic realm has been permanent in Austral biogeography (see [30,31]). At least for well-documented groups such as vascular plants, the existence of an “austral realm” has long been established [32], and more recently confirmed [11,33]. The terms Neantarctic, Holantarctic, Australasiatic, and Austral have similar meanings, emphasizing the biogeographical relationships of the disjunct distribution across the Pacific, mainly in southern South America and Australasia (New Zealand and Australia), with a minor relationship to the Cape Region in South Africa. Analyzing the distribution of vascular plants, Moreira-Muñoz [33] concluded that “there are 15 families and c. 60 genera that, under current taxonomic treatment, support the segregation of an Austral realm” [33] (p. 1657).

In Porter’s concept of ichneumonid biogeography, 39 genera (32%) belong to the endemic element; 31 genera (25%) correspond to the cosmopolitan element; 20 genera (16%) belong to the Holarctic–Oriental element; 17 genera (14%) correspond to the Neotropical element; just 4 genera comprise the Transantarctic (Australasian) element; and 3 genera belong to the disjunct Holarctic–Neotropical–Australasian element.

The four main elements—endemic, cosmopolitan, Holarctic–Oriental, and Neotropical—are recognizable according to current knowledge as follows: cosmopolitan element (n = 50, 36%), endemic element (n = 29, 21%), Neotropical element (n = 22, 16%), Holarctic–Oriental (n = 19, 14%). We also now recognize a south-temperate element (n = 16, 11%) and, following taxonomic updating, the Australasian element is now only composed of three genera: *Anacis* (Cryptinae), *Labena* (Labeninae) and *Meringops* (Phygadeuontinae).

According to Porter [15], these endemic genera sharply define the Neantarctic realm. The Neantarctic realm has a variety of landscapes ranging from semi-desert with sclerophyllous woods to humid *Nothofagus* forests in the south. It also has a disjunct relationship with certain genera in Australia and the Holarctic region, revealing the absence of dominant Neotropical taxa. In addition, Porter [15] divided the Neantarctic realm into four provinces according to the biota and phytophysiognomy of its environments: Atacamense; Mediterranean or Central; Valdivian; and Magallanic. The Valdivian province is the Chilean region with the greatest diversity of Neantarctic Ichneumonidae. This is because Darwin wasps prefer to frequent humid environments or close forests. After all, most species are hygrophilous and sylvatic (but, especially under cool thermal regimes, they also tend to invade open forests and grasslands) [16]. Valdivian vegetation is characterized by temperate evergreen forest, typically with neatly developed herbaceous, shrubby, and arboreal strata. The dominant tree niche is occupied mainly by various *Nothofagus*, *Eucryphia*, *Gevuina*, *Embothrium*, *Lomatia*, and *Drimys* species. This province is one of the best-defined centers of endemism in South America [15,34].

Moreira-Muñoz [11] says that it would be better to refer to the Neotropical element as an “American” element that ranges from the Northwestern United States to Mexico, Central America, the Northern Andes, Amazonia, the Central Andes, and the Southern Andes, including Chile and Argentina. However, Porter [15] emphasizes the fact that Chilean ichneumonid fauna differs from the rest of the Neotropics due to having the highest presence of endemic taxa and the lack of a predominance of Neotropical taxa in the Neantarctic realm south of 27 degrees [15,16]. According to Araujo et al. [19], six neotropical genera have widely distributed species in northern and southern Chile (*Dotocryptus*, *Trachysphyrus*, *Itamuton*, *Thymebatis*, *Alophophion*, and *Calliephialtes*), five genera are exclusive to Northern Chile (*Cyclaulus*, *Cosmiocryptus*, *Hypsanacis*, *Microcharops*, and *Carinodes*), three genera are exclusive to Central Chile (*Phycitiplex*, *Prochas*, and *Diacanthatius*), five genera are distributed in Central–Southern Chile (*Aeglocryptus*, *Neocryptopterys*, *Cecidopimpla*, *Diradops*, *Coelorhachis*, and *Stethantyx*), one neotropical genus (*Xylacis*) is exclusive to Southern Chile, and the genus *Odontopimpla* has uncertain distribution in Chile.

The Neotropical element can be further split into several subgroups (described as generalized tracks in Moreira-Muñoz [11]): Wide Neotropical track—composed of genera occurring in NW United States, from Mexico to Chile. Among Darwin wasps, seven neotropical genera were identified: *Diacantharius*, *Diradops*, *Microcharops*, *Carinodes*, *Calliephialtes*, *Odontopimpla*, and *Stethantyx*. Two genera (*Diacantharius* and *Odontopimpla*) reach Mexico but not the United States. Wide Andean track—composed of genera occurring in Costa Rica and ranging from Colombia to Chile. Five genera were identified: *Cecidopimpla*, *Dotocryptus*, *Trachysphyrus*, *Hypsanacis*, and *Alophophion*. Only *Cecidopimpla* reached Costa Rica. Central Andean or Altiplano track—composed of genera occurring in the Andean Altiplano in Peru, Chile, Bolivia, and Argentina. Five genera were identified: *Cyclaulus*, *Cosmiocryptus*, *Itamuton*, *Phycitiplex*, and *Xylacis*. South Amazonian track—composed of genera occurring in the Andes and southern Amazonia. Five genera were identified: *Prochas*, *Aeglocryptus*, *Neocryptopteryx*, *Thymebatis*, and *Alophophion*. According to Fernandes et al. [35], in Brazil these five genera are distributed mainly in the South, Southwest, and Northeast regions. *Phycitiplex* is only distributed in Argentina, Chile, and Uruguay.

Regarding the Holarctic–Oriental element, Porter [15] mentions that the Holarctic genera arrived in the Eocene and Oligocene eras, mainly because the Oligocene and subsequent periods experienced cold pulses (the world has undergone repeated changes from cold to tropical and from humid to arid climatic regimes since the Oligocene) that allowed for the exchange of Holarctic biota across the Andes and Central American mountains to the south and from the Andean–Patagonian regions to the north.

Australasian (or Transantarctic) genera present in the New World and Australia are very similar to the oldest insect orders (e.g., Ephemeroptera, Odonata, Plecoptera, Mecoptera), as they frequently appear among vascular plants [11]. According to the evidence, Labeninae radiated throughout Gondwana after the separation of Africa, India, and Madagascar, but before the separation of Australia [36]. This indicates a plausible pathway for biotic exchange between South America and Australia, possibly via Antarctica. Biogeographic inference also reveals that North American groups underwent more recent range expansions before the formation of the Isthmus of Panama land bridge [37]. This implies a more intricate scenario for Labeninae biogeography than previously anticipated.

Porter [15] already proposed that Chilean Darwin wasps have a connection to Antarctica. According to the hypothesis, the major lineages of this family already existed during the Cretaceous era, which was characterized by a much warmer and more humid global climate than today, and South America was still connected to Antarctica. During this period, a diverse biota evolved in Antarctica, which was extensively shared with southern America and Australia. Antarctica and South America remained connected until about 25 mya [38]. The unusual and endemic biota of Neantarctic South America probably survived by moving north from Antarctica before its glaciation. The mid-Cenozoic era marked the isolation of the Neantarctic realm along its eastern and northern boundaries due to mountain building, desertification, and cold climatic changes. As a result, the region’s insect biota has evolved in isolation for the last 25 million years (though more recent relations across the sea have also been proven) [39].

For the diversity analysis along the latitudinal gradient, we were able to plot occurrences for 264 species arranged in 102 genera, of which 65 were composed of just one species, which again indicates a depauperate fauna and/or knowledge gaps. Other regions such as Mexico also show a high proportion of genera (n = 123) composed of just one species [40].

In Chilean ichneumonid fauna, many species are represented by only one specimen, such as *Aeglocryptus nigricornis* (Brullé, 1846); *Acidnus ensifer* (Townes, 1970); *Aglaodina hyperbas* (Porter, 1967); *Barronia araucaria* (Gauld & Wahl, 2002), *Microcharops anticarsiae* (Gupta, 1987); and *Venturia porteri* (Brèthes, 1913). Others reach almost 20 species, such as *Nemeritis scaramozzinoi* Di Giovanni & Araujo, 2021 and *Trachysphyrus agenor* Porter, 1967, while others reach a dozen species, such as *Tycherus chileator* Diller, 2009 and *Trachysphyrus penai* Porter, 1967. Most genera are represented by few species, and these species are represented by few specimens. At this stage, it is not possible to discern between species that are indeed rare in the field from those that are under-collected due to limited collection efforts and a lack of specialists (a characteristic of the concept of “hidden biodiversity”).

A total of 264 species and 102 genera is not a large number compared to megadiverse countries such as Brazil or Mexico (with 1066/239 and 1031/373 species/genera, respectively). Mexico registers 45% endemic species, while Brazil registers just 3.1% endemic species [35,40].

Comparing the richness of Chilean Ichneumonidae with the biogeographical regions of Tenebrionidae in Chile proposed by Peña [41], as well as the more recent ecoregions proposed by Morrone [42], Darwin wasps are mainly found in the central regions of coastal Cordillera and southern Andean Cordillera [41], currently classified by Morrone [42] as the districts of Santiago Province (33–37° S) in the Central Chilean subregion and the Northern Valdivian Forest and Valdivian Forest regions [41]. Both regions are classified by Morrone [42] as provinces (in part) of the Subantarctic Region, with the Northern Valdivian Forest in Maule Province (37–39° S) and the Valdivian Forest in the Valdivian Forest Province (39–47° S).

The distribution pattern of Chilean Darwin wasps at the regional level does not support an increasing richness towards the tropics nor an increasing richness towards the south, with the highest richness maintained at mid-latitudes, as has been shown for comparable groups such as Chilean bees [43]. Chilean butterflies also display the highest richness at mid-latitudes [44].

This pattern supports the traditional (but debated) latitudinal trend of increasing richness towards the tropics. But in the case of ichneumonids, the taxonomic and distributional knowledge is still too limited (Wallacean and Linnean shortfalls); however, changes in this tendency can confidently be expected in the coming years (if more specialists join the challenge). The pattern is similar in comparable megadiverse botanical groups such as the Asteraceae; Chile harbors much less diversity than Brazil or Peru [45]. This is a consistent pattern in this biotic group, where the taxonomic and distributional knowledge is much more reliable.

In the case of Ichneumonidae, even if the Linnean and Wallacean shortfalls are better filled in the coming years, the richness pattern will continue to have a bimodal distribution and be concentrated at mid-latitudes due to the presence of the Atacama Desert, which constitutes a physical barrier for the diversification of life. This fact does not imply a merely barren environment, but an evolutionary arena favoring several groups such as Cactaceae [11] (p. 202) and Nolaneae [46] in the case of plant groups adapted to aridity.

The inverse latitudinal pattern of ichneumonids and the concentration at mid-latitudes, if confirmed for the Southern Hemisphere, still need to be tested regarding several explanations related to life history traits and attack strategies, as revised by Santos and Quicke [12]. Attack strategies are divided into different ovipositor lifestyles: idiobionts and koinobionts. According to Santos and Quicke [12] (and references therein), the concentration of the richness towards the south could be based on certain non-mutually exclusive explanations, such as (a) the resource fragmentation hypothesis, proposing that the diversity of hosts rises towards the equator, but that the density of each host population is too low to support koinobiont species; (b) the predation on hosts hypothesis, suggesting that predation on herbivores in the tropics is greater than in temperate regions; (c) the interphyletic competition hypothesis that parasitoids have to compete for hosts with other parasitic organisms that are more diverse in the tropics; and (d) the “nasty hosts hypothesis,” based on the tendency of tropical plants to have more chemical toxins than their temperate counterparts [12] (p. 374).

The concentration of Chilean ichneumonids at mid-latitudes is coincident with the highest presence of tree species at middle latitudes around 36° and 40° S, at the transition from the Mediterranean matorral to the temperate forest ecoregions [34]. This issue has been described as a “Gondwanan legacy” [47]. Nothofagus tree species, a potential habitat for parasitoid hosts, range from 33° S toward Cape Horn, but their richness is concentrated from 35° S to 42° S [11] (p. 256). Some studies note defoliator species such as the “sawfly from roble and raulí”, parasited by *Clasis* sp. [48].

Though species and generic richness are concentrated in several cells at mid-altitudes in the contact zone between the Valdivian Forests ecoregion and the Chilean Matorral, the need for additional sampling and the explicit assessment of collection biases and gaps through richness estimators is evident (see [49]).

Comparing altitudinal patterns, Flinte et al. [50] discovered that low- and mid-altitude areas on a mountain in the Brazilian Atlantic Forest contained significant diversity in terms of Darwin wasps, unlike their high-altitude counterparts. Moreover, distinct species were observed at various elevations along the mountain. These results imply that tropical forests could potentially host concentrated populations of Darwin wasps and deforestation poses a substantial risk of losing this biodiversity. Prioritizing the conservation of forests at low-to-middle altitudes may prove most effective in safeguarding the diversity of these wasps, though ensuring protection across a broad altitude range is essential for the preservation of all species.

**Figure 7:**
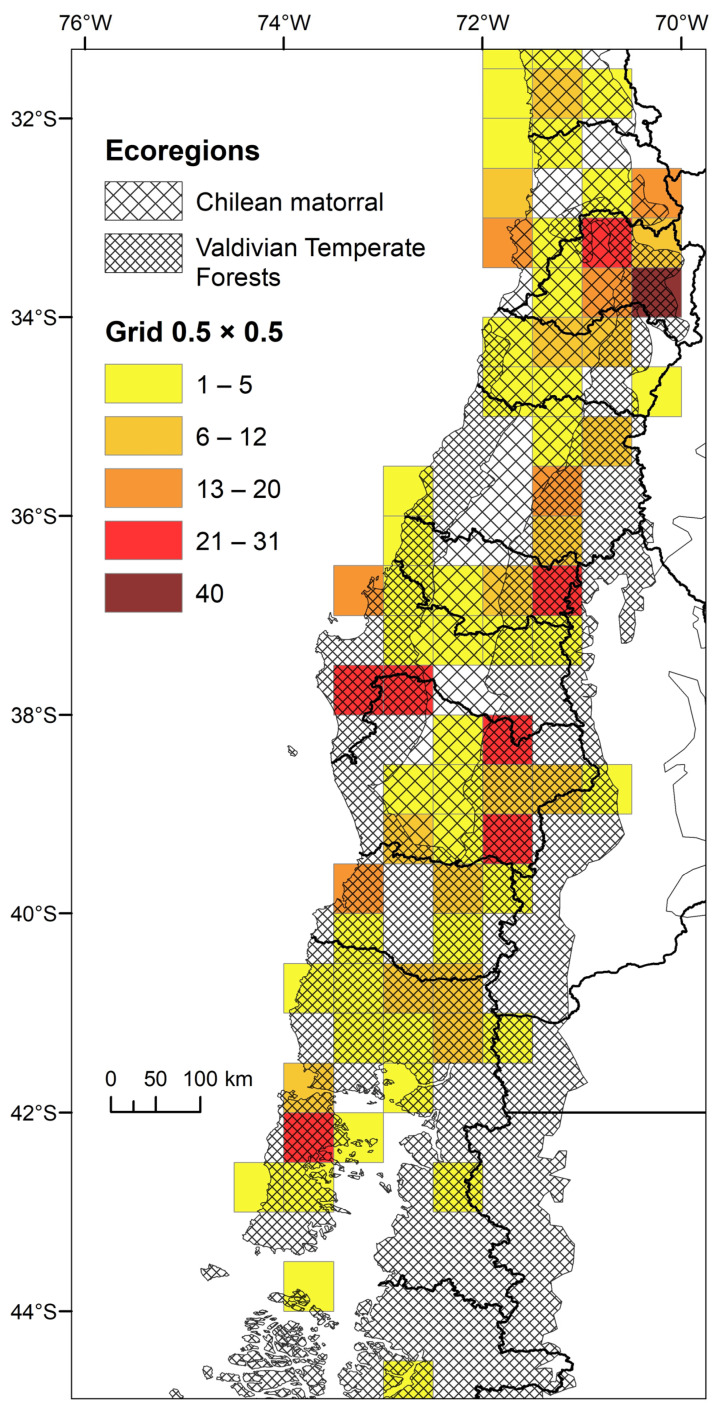
Species-level richness of Chilean ichneumonids is concentrated in mid-latitudes between 32° and 43° degrees south. Several cells (0.5 × 0.5 degrees) with the highest numbers occur in the contact zone between the Valdivian Forest and the Chilean Matorral ecoregions.

The knowledge of Chilean ichneumonid biodiversity richness is biased towards the most human-populated regions in the country. On the other hand, the highest levels of richness are in several cells in the contact zone between the Valdivian Forests ecoregion and the Chilean Matorral (Figure 7). It should be noted that several cells represent emblematic collection localities, such as the Santiago Range, the Nahuelbuta Coastal Range, the Araucanía Range, and Torres del Paine National Park in Magallanes. In contrast, the Atacama Desert shows little or no presence of Darwin wasps (Figure 5), which is to be expected due to the lack of a forest ecosystem rich in potential hosts and humidity. According to Porter [16], ichneumonids are generally restricted to forests or jungles (regions with frequent dew or rainfall), with semi-arid and arid zones being unfavorable for them and their hosts as, according to Townes [51], adult Darwin wasps need to drink water once a day (in the form of condensed dew on plant leaves). Meanwhile, Gauld [52] comments the following on the Costa Rican Darwin wasp: “Species-richness is generally greatest in forests or other humid areas, whilst there are relatively few species in more open, dry habitats […]” [52] (p. 25). This may be one of the reasons for the scant richness of Darwin wasps in the Chilean North.

Several authors have already emphasized that the global distributional knowledge of Ichneumonidae is too limited to be able to reach conclusions about latitudinal and altitudinal trends in the family [7,53]. Our results reinforce the still limited taxonomic and distributional information on Chilean subtropical and temperate ichneumonid fauna. This also has implications for the conservation of “hidden biodiversity” [54]. We also have a long way to go regarding understanding the relationships between parasitoids and their hosts (e.g., [55]) so we can better understand functions within and across ecosystems and latitudinal gradients.

## Figures and Tables

**Table 1:** Darwin wasp genera elements from Chile (n = 139) (see Section 2).

	Elements	Genera (No.)	%
1	Cosmopolitan	50	36
2	Endemic	29	21
3	Neotropical	22	16
4	Holarctic–Oriental	19	14
5	South-temperate	16	11
6	Australasian	3	2

## Data Availability

Details regarding the supporting data will be reported soon in the World Ichneumonidae Database. The data can also be requested directly from the corresponding author.

## Supplementary Materials

The following supporting information can be downloaded at: https://www.mdpi.com/article/10.3390/insects15060415/s1, Table S1: Chilean genera, ordered by Darwin wasp (Ichneumonidae) elements: (1) Cosmopolitan, (2) Endemic, (3) Neotropical, (4) Holarctic-Oriental, (5) South-temperate, (6) Australasian; Table S2: Chilean Ichneumonidae database [55,56,57,58,59,60,61,62,63,64,65,66,67,68,69,70,71,72,73,74,75,76,77,78,79,80,81,82,83,84,85,86,87,88,89,90,91,92,93,94,95,96,97,98,99,100,101,102,103,104,105,106,107,108,109,110,111,112,113,114,115,116,117].
