# Supplementary material for: Chilean Darwin Wasps (Ichneumonidae): Biogeographic Relationships and Distribution Patterns
Source: Insects. 2024 Jun 4;15(6):415. doi: 10.3390/insects15060415 (PMC11203931; doi:10.3390/insects15060415)
Supplement: Supplementary file 1 [file insects-15-00415-s001.zip › Table S1_Darwin wasps Elements_final.pdf]

Table S1. Chilean genera, ordered by Darwin wasp (Ichneumonidae) elements: (1) Cosmopolitan, (2) Endemic, (3) Neotropical, (4) Holarctic-Oriental, (5) South-temperate, (6) Australasian.

Subfamily	Genus	Global distribution	Element	Native spp.	Endemic spp.	Subfamily global distribution (Yu et al. 2016)
Anomaloninae	*Habronyx*	Australasian; Nearctic; Oriental; Eastern Palaearctic; Neotropical (Chile, Peru, Bolivia, Costa Rica, Ecuador, Mexico); Western Palaearctic; Europe; Oceanic	1	2	2	Afrotropical; Australasian; Estern Palaearctic; Europe; Nearctic; Neotropical; Oceanic; Oriental; Western Palaearctic
Anomaloninae	*Parania*	Afrotropical; Europe, Nearctic; Oriental; Neotropical (Argentina, Brazil, Chile, Costa Rica, Paraguay, Uruguay); Western Palaearctic	1	?	?	–
Anomaloninae	*Therion*	Australasian; Eastern Palaearctic; Europe; Nearctic; Neotropical (Argentina, Bolivia, Chile, Panama, Mexico); Western Palaearctic; Oceanic	1	?	?	Afrotropical; Australasian; Estern Palaearctic; Europe; Nearctic; Neotropical; Oceanic; Oriental;

						Western Palaearctic
Banchinae	*Archoprotus*	Endemic (Chile)	2	1	1	Afrotropical; Australasian; Estern Palaearctic; Europe; Nearctic; Neotropical; Oceanic; Oriental; Western Palaearctic
Banchinae	*Exetastes*	Afrotropical; Eastern Palaearctic; Europe; Nearctic; Oriental; Neotropical (Brazil, Chile, Cuba, Mexico); Western Palaearctic	1	?	?	_
Banchinae	*Cecidopimpla*	Neotropical (Argentina, Brazil, Chile, Costa Rica)	3	?	?	_
Banchinae	*Diradops*	Nearctic; Neotropical (Brazil, Costa Rica, Chile, Guyana, Mexico, Panama, Venezuela)	3	?	?	_
Banchinae	*Lissonota*	Afrotropical; Australasian; Neartic; Oriental; Eastern Palaearctic; Neotropical (Chile, Costa Rica, Cuba, Ecuador, Mexico, Panama); Western Palaearctic; Europe; Oceanic	1	2	1	_
Banchinae	*Notostilbops*	Endemic (Chile)	2	1	1	___
Banchinae	*Pristiboea*	Endemic (Chile)	2	1	1	_
Banchinae	*Geraldus*	South-temperate (Argentina, Chile)	5	2	1	_

Banchinae	*Glypta*	Eastern Palaearctic; Neotropical (Argentina, Chile, Costa Rica, Mexico, Uruguay); Europe; Oriental; Nearctic; Western Palaearctic	4	2	2	–
Banchinae	*Valdiviglypta*	Endemic (Chile)	2	1	1	–
Brachycyrtinae	*Brachycyrtus*	Afrotropical; Australasian; Neartic; Oriental; Eastern Palaearctic; Neotropical (Argentina, Brazil, Chile, Costa Rica, Ecuador, Mexico, Panama, Peru, Suriname, Trinidad & Tobago, Venezuela); Western Palaearctic; Europe; Oceanic	1	?	?	Afrotropical; Australasian; Neartic; Oriental; Eastern Palaearctic; Neotropical; Western Palaearctic; Europe; Oceanic
Campopleginae	*Campoletis*	Afrotropical; Australasian; Neartic; Oriental; Eastern Palaearctic; Neotropical (Argentina, Barbados, Brazil, Chile, Mexico, Nicaragua, Peru, Uruguay); Western Palaearctic; Europe; Oceanic	1	1	0	Afrotropical; Australasian; Estern Palaearctic; Europe; Nearctic; Neotropical; Oceanic; Oriental; Western Palaearctic
Campopleginae	*Campoplex*	Afrotropical; Australasian; Neartic; Oriental; Eastern Palaearctic; Neotropical (Argentina, Brazil, Chile, Guyana, St. Vicent, Uruguay); Western Palaearctic; Europe; Oceanic	1	7	7	–

Campopleginae	*Diadegma*	Afrotropical; Australasian; Neartic; Oriental; Eastern Palaearctic; Neotropical (Argentina, Brazil, Chile, Colombia, Cuba, Honduras, Jamaica, Mexico, Peru, Uruguay, Venezuela); Western Palaearctic; Europe; Oceanic	1	4	2	–
Campopleginae	*Hyposoter*	Afrotropical; Australasian; Neartic; Oriental; Eastern Palaearctic; Neotropical (Argentina, Brazil, Chile, Cuba, Mexico, Juan Fernandez Islands); Western Palaearctic; Europe; Oceanic	1	2	2	–
Campopleginae	*Microcharops*	Eastern Palaearctic; Nearctic; Neotropical (Argentina, Brazil, Bolivia, Colombia, Cuba, Honduras, Mexico, Peru, Panama, Suriname, Venezuela, Trinidad & Tobago, Puerto Rico, Paraguay, Nicaragua, Jamaica, Guyana, Ecuador, Costa Rica, Chile, Grenada)	4	1	0	–
Campopleginae	*Nemeritis*	Eastern Palaearctic; Oriental; Europe; Western Palaearctic; Nearctic; Neotropical (Chile)	4	1	1	–
Campopleginae	*Venturia*	Afrotropical; Australasian; Neartic; Oriental; Eastern Palaearctic; Neotropical (Argentina, Bolivia, Brazil, Chile, Colombia, Costa Rica, Cuba, Guatemala, Mexico, Panama, Paraguay, Peru, Puerto Rico, St. Vicent, Suriname, Uruguay,	1	1	1	–

		Venezuela); Western Palaearctic; Europe; Oceanic				
Campopleginae	*Casinaria*	Afrotropical; Australasian; Neartic; Oriental; Eastern Palaearctic; Neotropical (Argentina, Brazil, Chile, Cuba, Costa Rica Ecuador, Mexico, Peru, Uruguay); Western Palaearctic; Europe; Oceanic	1	?	?	–
Campopleginae	*Cymodusa*	Eastern Palaearctic; Europe; Nearctic; Neotropical (Chile, Ecuador, Mexico, Panama); Oriental; Western Palaearctic	4	?	?	–
Campopleginae	*Dusona*	Afrotropical; Australasian; Neartic; Oriental; Eastern Palaearctic; Neotropical (Argentina, Brazil, Chile, Cuba, Guyana, Mexico, Paraguay, Peru); Western Palaearctic; Europe; Oceanic	1	?	?	–
Campopleginae	*Phobocampe*	Nearctic; Oriental; Eastern Palaearctic; Neotropical (Chile, Mexico); Western Palaearctic; Europe	4	?	?	–
Campopleginae	*Campoctonus*	Nearctic, Neotropical (Argentina, Brazil, Chile, Mexico)	4	?	?	–
Campopleginae	*Meloboris*	Afrotropical; Australasian; Estern Palaearctic; Europe; Nearctic; Neotropical (Chile, Mexico); Oceanic; Western Palaearctic	1	?	?	–
Campopleginae	*Prochas*	Neotropical (Brazil, Chile, Trinidad & Tobago)	3	?	?	–

Claseinae	*Clasis*	Endemic (Chile, Juan Fernandez Islands)	2	1	1	Neotropical
Claseinae	*Ecphysis*	Endemic (Chile)	2	1	1	–
Cremastinae	*Pristomerus*	Afrotropical; Australasian; Eastern Palaearctic; Europe; Nearctic; Neotropical (Argentina, Brazil, Chile, Costa Rica, Guatemala, Guyana, Honduras, Mexico); Oceanic; Oriental; Western Palaearctic	1	1	1	Afrotropical; Australasian; Estern Palaearctic; Europe; Nearctic; Neotropical; Oceanic; Oriental; Western Palaearctic
Cremastinae	*Trathala*	Afrotropical; Australasian; Estern Palaearctic; Europe; Nearctic; Neotropical (Argentina, Bolivia, Chile, Costa Rica, El Salvador, Mexico); Oceanic; Oriental; Western Palaearctic	1	?	?	–
Cryptinae	*Cryptus*	Afrotropical; Eastern Palaearctic; Europe; Nearctic; Neotropical (Brazil, Chile, Costa Rica, Mexico); Oriental; Western Palaearctic	1	5	5	Afrotropical; Australasian; Estern Palaearctic; Europe; Nearctic; Neotropical; Oceanic; Oriental; Western Palaearctic

Cryptinae	*Dotocryptus*	Neotropical (Argentina, Brazil, Chile, Ecuador, Paraguay, Colombia)	3	2	0	–
Cryptinae	*Trachysphyrus*	Neotropical (Argentina, Bolivia, Brazil, Chile, Colombia, Ecuador, Paraguay, Peru, Uruguay)	3	13	7	–
Cryptinae	*Caenopelte*	Endemic (Chile)	2	1	1	–
Cryptinae	*Cyclaulus*	Neotropical (Argentina, Chile, Peru)	3	1	0	–
Cryptinae	*Aeglocryptus*	Neotropical (Argentina, Brazil, Chile, Peru, Uruguay)	3	2	0	–
Cryptinae	*Aglaodina*	South-temperate (Argentina, Chile)	5	2	1	–
Cryptinae	*Anacis*	Australasian; Neotropical (Argentina, Chile, Venezuela, Ecuador, Peru)	6	7	2	–
Cryptinae	*Araucacis*	Endemic (Chile)	2	2	2	–
Cryptinae	*Chilecryptus*	South-temperate (Argentina, Chile)	5	4	2	–
Cryptinae	*Cosmiocryptus*	Neotropical (Argentina, Chile, Peru)	3	2	1	–
Cryptinae	*Hypsanacis*	Neotropical (Argentina, Chile, Ecuador)	3	1	1	–
Cryptinae	*Itamuton*	Neotropical (Argentina, Chile, Peru)	3	3	1	–
Cryptinae	*Mesostenus*	Eastern Palaearctic; Western Palaearctic; Neotropical (Argentina, Bolivia, Brazil, Chile, Mexico, Peru, Venezuela); Europe; Nearctic; Oriental; Oceanic	1	1	1	–
Cryptinae	*Myrmecacis*	South-temperate (Argentina, Brazil, Chile)	5	1	1	–
Cryptinae	*Neocryptopteryx*	Neotropical (Argentina, Brazil, Chile, Peru, Uruguay)	3	4	2	–

Cryptinae	*Nothischnus*	Endemic (Chile)	2	1	1	_
Cryptinae	*Oecetiplex*	South-temperate (Argentina, Chile)	5	1	0	_
Cryptinae	*Periplasma*	Endemic (Chile)	2	1	1	_
Cryptinae	*Phycitiplex*	Neotropical (Argentina, Chile, Uruguay)	3	1	1	_
Cryptinae	*Picrocryptoides*	South-temperate (Argentina, Chile)	5	1	1	_
Cryptinae	*Sciocryptus*	South-temperate (Argentina, Chile)	5	1	0	_
Cryptinae	*Xiphonychidion*	South-temperate (Argentina, Chile)	5	7	3	_
Cryptinae	*Xylacis*	Neotropical (Argentina, Bolivia, Chile)	3	1	0	_
Ctenopelmatinae	*Cataptygma*	South-temperate (Argentina, Chile)	5	3	2	Afrotropical; Australasian; Estern Palaearctic; Europe; Nearctic; Neotropical; Oceanic; Oriental; Western Palaearctic
Ctenopelmatinae	*Pedinopa*	Endemic (Chile)	2	1	1	_
Ctenopelmatinae	*Tetrambon*	South-temperate (Argentina, Chile)	5	2	2	_
Ctenopelmatinae	*Cacomisthus*	Endemic (Chile)	2	1	1	_
Ctenopelmatinae	*Petilium*	Endemic (Chile)	2	2	2	_
Ctenopelmatinae	*Stipomoles*	Endemic (Chile)	2	1	1	_
Ctenopelmatinae	*Coelorhachis*	Neotropical (Chile, Costa Rica, Guatemala, Mexico)	3	?	?	_
Diplazontinae	*Diplazon*	Afrotropical; Australasian; Eastern Palaearctic; Europe; Nearctic;	1	2	0	Afrotropical; Australasian;

		Neotropical (Argentina, Brazil, Chile, Costa Rica, Guatemala, Mexico, Peru, Uruguay); Oceanic; Oriental; Western Palaearctic				Estern Palaearctic; Europe; Nearctic; Neotropical; Oceanic; Oriental; Western Palaearctic
Diplazontinae	*Sussaba*	Eastern Palaearctic; Europe; Nearctic; Neotropical (Argentina, Chile, Colombia Costa Rica, Ecuador); Oriental; Western Palaearctic	4	5	5	–
Diplazontinae	*Syrphoctonus*	Afrotropical; Australasian; Eastern Palaearctic; Europe; Nearctic; Neotropical (Argentina, Bolivia, Brazil, Chile, Colombia, Costa Rica, Ecuador, Mexico, Peru, Venezuela); Oceanic; Oriental; Western Palaearctic	1	9	8	–
Diplazontinae	*Woldstedtius*	Australasian; Eastern Palaearctic; Europe; Nearctic; Neotropical (Bolivia, Chile, Colombia, Costa Rica, Ecuador, Mexico, Peru); Oceanic; Oriental; Western Palaearctic	1	2	2	–
Eucerotinae	*Barronia*	Endemic (Chile)	2	1	1	Afrotropical; Australasian; Estern Palaearctic;

						Europe; Nearctic; Neotropical; Oceanic; Oriental; Western Palaearctic
Ichneumoninae	*Carinodes*	Nearctic; Neotropical (Brazil, Chile, Cuba, Guatemala, Guyana, Mexico, Panama, Peru, Puerto Rico)	3	2	2	Afrotropical; Australasian; Estern Palaearctic; Europe; Nearctic; Neotropical; Oceanic; Oriental; Western Palaearctic
Ichneumoninae	*Hoplismenus*	Australasian; Eastern Palaearctic; Europe; Nearctic; Neotropical (Chile, Mexico); Oriental; Western Palaearctic	1	1	1	–
Ichneumoninae	*Ichneumon*	Afrotropical; Australasian; Eastern Palaearctic; Europe; Nearctic; Neotropical (Chile, Cuba, Ecuador, Guatemala, Mexico, Suriname); Oceanic; Oriental; Western Palaearctic	1	3	3	–
Ichneumoninae	*Setanta*	Australasian; Eastern Palaearctic; Nearctic; Neotropical (Argentina,	1	1	0	–

		Chile, Costa Rica, Guatemala, Mexico); Oriental				
Ichneumoninae	*Stenobarichneumon*	Eastern Palaearctic; Europe; Nearctic; Neotropical (Chile); Western Palaearctic	4	1	1	_
Ichneumoninae	*Thymebatis*	Neotropical (Argentina, Brazil, Chile, Juan Fernandez Islands, Uruguay)	3	7	5	_
Ichneumoninae	*Dicaelotus*	Afrotropical; Eastern Palaearctic; Europe; Nearctic; Neotropical (Bolivia, Brazil, Chile, Ecuador, Mexico); Western Palaearctic	1	1	1	_
Ichneumoninae	*Tycherus*	Afrotropical; Eastern Palaearctic; Europe; Nearctic; Neotropical (Argentina, Brazil, Chile, Mexico); Oriental; Western Palaearctic	1	35	35	_
Ichneumoninae	*Platylabus*	Afrotropical; Australasian; Eastern Palaearctic; Europe; Nearctic; Neotropical (Chile, Guatemala, Mexico); Oriental; Western Palaearctic	1	1	1	_
Ichneumoninae	*Barythixis*	South-temperate (Argentina, Chile)	5	1	0	_
Ichneumoninae	*Diacantharius*	Neotropical (Argentina, Brazil, Chile, Guatemala, Mexico, Peru)	3	?	?	_
Ichneumoninae	*Chilelabus*	Endemic (Chile)	2	1	1	_
Ichneumoninae	*Chilhoplites*	South-temperate (Argentina, Chile)	5	1	1	_
Ichneumoninae	*Ithaechma*	Endemic (Chile)	2	1	1	_
Ichneumoninae	*Notophasma*	South-temperate (Argentina, Chile)	5	1	0	_
Ichneumoninae	*Zophoplites*	Endemic (Chile)	2	1	1	_

Ichneumoninae	*Melanichneumon*	Australasian; Estern Palaearctic; Europe; Nearctic; Neotropical (Chile, Mexico); Oriental; Western Palaearctic	1	?	?	–
Ichneumoninae	*Eutanyacra*	Australasian; Estern Palaearctic; Europe; Nearctic; Neotropical (Chile, Mexico); Oriental; Oceanic; Western Palaearctic	1	?	?	–
Ichneumoninae	*Diphyus*	Afrotropical; Estern Palaearctic; Europe; Nearctic; Neotropical (Chile, Costa Rica, Guatemala, Mexico); Oriental; Oceanic; Western Palaearctic	1	?	?	–
Labeninae	*Gauldianus*	Endemic (Chile)	2	1	1	Australasian; Nearctic; Neotropical; Oceanic
Labeninae	*Labena*	Australasian, Nearctic, Neotropical (Argentina, Brazil, Chile, Colombia, Costa Rica, French Guiana, Guatemala, Guyana, Mexico, Panama, Paraguay, Peru), Oceanic	6	2	2	–
Labeninae	*Torquinsha*	South-temperate (Argentina, Chile)	5	2	0	–
Labeninae	*Grotea*	Nearctic; Neotropical (Argentina, Belize, Brazil, Chile, Colombia, Costa Rica, Ecuador, Guyana, Leewards Islands, Mexico, Panama, Peru)	3	6	4	–
Mesochorinae	*Chineater*	Endemic (Chile)	2	1	1	Afrotropical; Australasian; Estern

						Palaearctic; Europe; Nearctic; Neotropical; Oceanic; Oriental; Western Palaearctic
Mesochorinae	*Cidaphus*	Afrotropical; Australasian; Eastern Palaearctic; Europe; Nearctic; Neotropical (Bolivia, Brazil, Chile, Mexico, Peru); Oriental; Western Palaearctic	1	1	1	–
Mesochorinae	*Latilumbus*	Endemic (Chile)	2	1	1	–
Mesochorinae	*Lepidura*	South-temperate (Argentina, Chile)	5	13	10	–
Mesochorinae	*Mesochorus*	Afrotropical; Australasian; Eastern Palaearctic; Europe; Nearctic; Neotropical (Argentina, Bolivia, Brazil, Chile, Colombia, Costa Rica, Cuba, Ecuador, El Salvador, Guatemala, Guyana, Honduras, Jamaica, Mexico, Nicaragua, Panama, Paraguay, Peru, Suriname, Venezuela); Oceanic; Oriental; Western Palaearctic	1	20	13	–
Metopiinae	*Colpotrochia*	Australasian; Eastern Palaearctic; Europe; Nearctic; Neotropical (Brazil, Chile, Costa Rica, Cuba, Mexico, Paraguay, Puerto Rico); Oriental; Western Palaearctic	1	1	1	Afrotropical; Australasian; Estern Palaearctic; Europe; Nearctic;

						Neotropical; Oceanic; Oriental; Western Palaearctic
Metopiinae	*Hypsicera*	Afrotropical; Australasian; Eastern Palaearctic; Europe; Nearctic; Neotropical (Argentina, Brazil, Chile); Oceanic; Oriental; Western Palaearctic	1	1	0	–
Metopiinae	*Scolomus*	Europe; Nearctic; Neotropical (Argentina, Chile, Costa Rica); Western Palaearctic	4	3	3	–
Metopiinae	*Seticornuta*	Eastern Palaearctic; Nearctic; Neotropical (Brazil, Chile, Costa Rica); Oriental	4	1	1	–
Ophioninae	*Alophophion*	Neotropical (Argentina, Bolivia, Brazil, Chile, Ecuador, Falkland Islands, Peru)	3	21	10	Afrotropical; Australasian; Estern Palaearctic; Europe; Nearctic; Neotropical; Oceanic; Oriental; Western Palaearctic
Ophioninae	*Enicospilus*	Afrotropical; Australasian; Eastern Palaearctic; Europe; Nearctic; Neotropical (Argentina, Bolivia, Belize, Brazil, Chile, Colombia,	1	4	0	–

		Costa Rica, Cuba, Ecuador, El Salvador, French Guiana, Guatemala, Guyana, Haiti, Honduras, Jamaica, Nicaragua, Panama, Paraguay, Peru, Puerto Rico, St. Vicente, Suriname, Trinidad & Tobago, Uruguay, Venezuela); Oceanic; Oriental; Western Palaearctic				
Ophioninae	*Ophion*	Australasian; Eastern Palaearctic; Europe; Nearctic; Neotropical (Argentina, Bolivia, Brazil, Chile, Cuba, Guatemala, Honduras, Mexico, Nicaragua, Panama, Paraguay, Peru, Puerto Rico, Uruguay, Venezuela); Oceanic; Oriental; Western Palaearctic	1	2	1	_
Orthocentrinae	*Megastylus*	Afrotropical; Estern Palaearctic; Europe; Nearctic; Neotropical (Chile, Mexico); Oceanic; Oriental; Western Palaearctic	1	?	?	Australasian; Estern Palaearctic; Europe; Nearctic; Neotropical; Oceanic; Western Palaearctic
Orthocentrinae	*Apoclima*	Estern Palaearctic; Europe; Nearctic; Neotropical (Chile, Mexico); Western Palaearctic	4	?	?	_

Orthocentrinae	*Helictes*	Estern Palaearctic; Europe; Nearctic; Neotropical (Chile, Mexico); Western Palaearctic	4	?	?	_
Orthocentrinae	*Symplecis*	Afrotropical; Estern Palaearctic; Europe; Nearctic; Neotropical (Chile, Mexico); Oriental; Western Palaearctic	1	?	?	_
Orthocentrinae	*Gnathochorisis*	Estern Palaearctic; Europe; Nearctic; Neotropical (Chile, Mexico); Western Palaearctic	4	?	?	_
Orthocentrinae	*Stenomacrus*	Australasian; Estern Palaearctic; Europe; Nearctic; Neotropical (Chile, Grenade, St. Vicent); Oceanic; Western Palaearctic	1	?	?	_
Pedunculinae	*Pedunculus*	Endemic (Chile)	2	1	1	Australasian; Neotropical
Phygadeuontinae	*Acidnus*	Endemic (Chile)	2	1	1	Afrotropical; Australasian; Estern Palaearctic; Europe; Nearctic; Neotropical; Oceanic; Oriental; Western Palaearctic
Phygadeuontinae	*Rhabdosis*	Endemic (Chile)	2	1	1	_
Phygadeuontinae	*Aclastus*	Estern Palaearctic; Europe; Nearctic; Neotropical (Chile, Peru); Oriental; Western Palaearctic	4	?	?	_

Phygadeuontinae	*Ethelurgus*	Estern Palaearctic; Europe; Nearctic; Neotropical (Argentina, Brazil, Chile, Colombia, Honduras, Mexico); Oriental; Western Palaearctic	4	?	?	_
Phygadeuontinae	*Surculus*	Endemic (Chile)	2	1	1	_
Phygadeuontinae	*Isdromas*	Estern Palaearctic; Europe; Nearctic; Neotropical (Argentina, Brazil, Chile, Colombia, Honduras, Mexico); Oriental; Western Palaearctic	1	?	?	_
Phygadeuontinae	*Meringops*	Australasian; Neotropical (Chile)	6	1	1	_
Phygadeuontinae	*Dichrogaster*	Afrotropical; Australasian; Eastern Palaearctic; Europe; Nearctic; Neotropical (Argentina, Chile, Brazil, Ecuador, Venezuela); Oriental; Western Palaearctic	1	1	1	_
Phygadeuontinae	*Gelis*	Afrotropical; Eastern Palaearctic; Europe; Nearctic; Neotropical (Argentina, Chile); Oceanic; Oriental; Western Palaearctic	1	1	1	_
Phygadeuontinae	*Peumocryptus*	Endemic (Chile)	2	1	1	_
Phygadeuontinae	*Xenolytus*	Afrotropical; Australasian; Eastern Palaearctic; Europe; Nearctic; Neotropical (Chile); Oceanic; Oriental; Western Palaearctic	1	1	0	_
Phygadeuontinae	*Bilira*	Endemic (Chile)	2	1	1	_
Phygadeuontinae	*Charitopes*	Afrotropical; Eastern Palaearctic; Europe; Nearctic; Neotropical (Argentina, Chile, Ecuador, Mexico,	1	1	0	_

		Peru, Venezuela); Oriental; Western Palaearctic				
Phygadeuontinae	*Distathma*	Eastern Palaearctic; Neotropical (Argentina, Brazil, Chile, Juan Fernandez Islands, Mexico); Oriental	4	4	2	–
Phygadeuontinae	*Teluncus*	Endemic (Chile)	2	1	1	–
Phygadeuontinae	*Atractodes*	Afrotropical; Eastern Palaearctic; Europe; Nearctic; Neotropical (Chile); Oriental; Western Palaearctic	1	2	2	–
Phygadeuontinae	*Stilpnus*	Eastern Palaearctic; Europe; Nearctic; Neotropical (Chile, Juan Fernandez Islands); Oriental; Western Palaearctic	4	1	0	–
Pimplinae	*Calliephialtes*	Nearctic; Neotropical (Argentina, Brazil, Chile, Costa Rica, Cuba, El Salvador, Mexico, Puerto Rico, Uruguay); Oceanic	3	2	1	Afrotropical; Australasian; Estern Palaearctic; Europe; Nearctic; Neotropical; Oceanic; Oriental; Western Palaearctic
Pimplinae	*Clistopyga*	Afrotropical; Eastern Palaearctic; Europe; Nearctic; Neotropical (Argentina, Bolivia, Brazil, Chile, Colombia, Costa Rica, Ecuador, El Salvador, French Guiana, Mexico,	1	1	1	–

		Paraguay, Peru, Venezuela); Oriental; Western Palaearctic				
Pimplinae	*Liotryphon*	Estern Palaearctic; Europe; Nearctic; Neotropical (Chile, Mexico); Oceanic; Oriental; Western Palaearctic	4	?	?	–
Pimplinae	*Odontopimpla*	Neotropical (Brazil, Chile, Costa Rica, Guatemala, Guyana, Honduras, Mexico, Nicaragua, Peru)	3	1	0	–
Pimplinae	*Tromatobia*	Afrotropical; Australasian; Eastern Palaearctic; Europe; Nearctic; Neotropical (Argentina, Bolivia, Chile, Brazil, Colombia, Costa Rica, Cuba, Mexico, Peru, Ouerto Rico, Uruguay, Venezuela); Oceanic; Oriental; Western Palaearctic	1	2	1	–
Pimplinae	*Itoplectis*	Afrotropical; Australasian; Eastern Palaearctic; Europe; Nearctic; Neotropical (Argentina, Bolivia, Brazil, Chile, Colombia, Costa Rica, Ecuador, Guyana, Mexico, Panama, Peru, Uruguay); Oceanic; Oriental; Western Palaearctic	1	1	0	–
Pimplinae	*Pimpla*	Afrotropical; Australasian; Eastern Palaearctic; Europe; Nearctic; Neotropical (Argentina, Bolivia, Brazil, Chile, Colombia, Costa Rica, Cuba, Ecuador, Guatemala, Haiti, Honduras, Jamaica, Mexico, Nicaragua, Panama, Paraguay, Peru, Puerto Rico, Uruguay, Venezuela);	1	5	1	–

		Oceanic; Oriental; Western Palaearctic				
Pimplinae	*Polysphincta*	Estern Palaearctic; Europe; Nearctic; Neotropical (Argentina, Belize, Brazil, Chile, Mexico); Oriental; Western Palaearctic	4	?	?	–
Tatogastrinae	*Tatogaster*	South-temperate (Argentina, Chile)	5	1	1	Neotropical
Tersilochinae	*Notophrudus*	Endemic (Chile)	2	1	1	Afrotropical; Australasian; Estern Palaearctic; Europe; Nearctic; Neotropical; Oceanic; Oriental; Western Palaearctic
Tersilochinae	*Stethantyx*	Nearctic; Neotropical (Argentina, Brazil, Chile, Colombia, Costa Rica, Ecuador, Mexico, Paraguay, Peru, Suriname, Trinidad & Tobago, Uruguay)	3	?	?	–
Tryphoninae	*Netelia*	Afrotropical; Australasian; Eastern Palaearctic; Europe; Nearctic; Neotropical (Argentina, Bolivia, Brazil, Chile, Colombia, Costa Rica, Cuba, Ecuador, Guatemala, Mexico, Nicaragua, Panama, Peru, Venezuela); Oceanic; Oriental; Western Palaearctic	1	3	0	Afrotropical; Australasian; Estern Palaearctic; Europe; Nearctic; Neotropical; Oceanic;

						Oriental; Western Palaearctic
